# Physical activity for people with young-onset dementia and carers: protocol for a scoping review

**DOI:** 10.1186/s13643-018-0698-5

**Published:** 2018-02-27

**Authors:** Chloe Rodgers, David Rogerson, Judy Stevenson, Davina Porock

**Affiliations:** 1 Academy of Sport and Physical activity, Sheffield Hallam University, Sheffield, UK; 2 Faculty of Health and Wellbeing, Sheffield Hallam University, Sheffield, UK; 3 A214 Collegiate Hall, Collegiate Crescent, Sheffield Hallam University, Sheffield, S10 2BP UK

**Keywords:** Physical activity, Exercise, Young-onset dementia, Carers

## Abstract

**Background:**

Physical activity has been cited as a potential symptomatic treatment option for people living with dementia. At present, much of the research concerning physical activity and dementia considers older adults, and there are several review articles summarising the evidence in this area. Less is known about physical activity for younger people with dementia, despite the marked differences in needs and preferences between the two groups. The aim of this scoping review is to systematically explore and critically appraise the current state of the evidence regarding physical activity for people with young-onset dementia and carers.

**Methods:**

Several electronic databases (i.e. MEDLINE, SPORTDiscus, CINAHL, Cochrane Library, PsycINFO, Applied Social Sciences Index & Abstracts (ASSIA) and Scopus), grey literature (i.e. NICE Evidence Search (UK) and targeted international organisations e.g. Alzheimer’s Society (UK), Age UK, Young Dementia UK, Alzheimer’s Association (USA), Dementia Australia) and trial registries (i.e. UK Clinical Trials Gateway, International Clinical Trials Registry Platform and EU Clinical Trials Register) will be searched for published and unpublished evidence regarding physical activity for people with young-onset dementia and carers. Studies included in the review will be subjected to a narrative synthesis to explore similarities and differences, both within and between studies, to identify patterns and themes and to postulate explanations for research findings (e.g. how and why certain interventions or programmes have worked (or not); factors that might have influenced the findings ).

**Discussion:**

This will be the first review to systematically explore and critically appraise the current state of the evidence regarding physical activity for people with young-onset dementia and carers. It is hoped that findings from this review will be used to inform the development of future physical activity interventions, to serve as a basis for consultation with key stakeholders and to identify appropriate outcome measures relevant to people with young-onset dementia and carers.

**Systematic review registration:**

At present, scoping reviews are not eligible for registration on the international prospective register of systematic reviews (i.e. PROSPERO).

**Electronic supplementary material:**

The online version of this article (10.1186/s13643-018-0698-5) contains supplementary material, which is available to authorized users.

## Background

Dementia is an umbrella term used to describe a range of neurodegenerative diseases, of which Alzheimer’s disease is the most well-known [1]. Dementia is a major neurocognitive disorder, in which the primary clinical symptom is a reduction in cognitive function compared to previous levels that is sufficient to interfere with a person’s ability to perform everyday tasks independently [2]. Young-onset dementia, defined as symptoms of dementia presenting before the age of 65 years, accounts for approximately 5% of all dementia cases in the UK (*n* = 42,500) [3]. Young-onset dementia differs from late-onset dementia and is not simply the same disease occurring in younger populations. Younger people have more heterogeneous diagnoses (i.e. fewer cases of Alzheimer’s disease and more cases of frontotemporal dementia) [4], are more likely to have familial forms of dementia (e.g. autosomal dominant Alzheimer’s disease) [5] and may have atypical disease presentations (e.g. non-memory Alzheimer’s disease phenotype) [6]. Younger people with dementia may also experience different psychosocial challenges compared to those diagnosed in later life, such as coping with an unexpected decline in health during midlife, loss of employment, caring for children and/or older relatives and managing changes in spousal/familial relationships [7].

At present, there is no known cure for dementia. As such, interventions to slow progression and to attenuate symptoms are of particular interest. Non-pharmacological interventions, such as physical activity, are gaining recognition as possible symptomatic treatment options [8]. Regular physical activity has known health benefits for most people, including reduced all-cause mortality, improved cardiovascular health and reduced incidence of obesity [9]. Physical activity may also improve physical, psychological and social health outcomes in people living with dementia [10]. A meta-analysis of 18 randomised control trials found that physical activity improved both cognitive function and ability to undertake activities of daily living in people with dementia [11]. In contrast, however, a recent Cochrane Review of 17 randomised control trials found insufficient evidence to suggest that physical activity improved cognition, neuropsychiatric symptoms (e.g. aggression and wandering) or depression in people with dementia but that it may improve activities of daily living [12]. The authors suggest that further research is needed to identify the optimum physical activity intervention (i.e. frequency, intensity, duration of activity). Of note, all of the aforementioned reviews consider physical activity for older people with dementia [10–12], and to date, no review has specifically focussed on younger people, despite the marked differences in needs and preferences between the two groups [13].

By definition, younger people with dementia are of working age at the point of diagnosis, and thus, an abrupt end to employment may impact their sense of identity (e.g. ‘breadwinner’ to dependant), social relationships (e.g. work colleagues becoming distant) and feelings of independence, self-worth and purpose in life [14]. Narratives from younger people with dementia reveal that most are aware of the decline in their capacity and the impact of this on family and friends [15]. This may explain the high incidence of depression reported in this group [16]. In general, younger people with dementia are more physically fit [17] and tend to live with fewer comorbidities than those diagnosed in later life [18]. As such, younger people may not be well suited to join physical activity programmes designed for older adults. Indeed, a lack of common interests with older people and a desire to socialise with people of a similar age have been cited as factors influencing service use by younger people with dementia [17, 19]. Public health interventions should be underpinned by evidence, and therefore, a comprehensive and explorative review of the current evidence to inform the development of future physical activity interventions for younger people with dementia and to identify outcomes that are relevant to this group is warranted.

Despite recent research interest around the potential benefits of physical activity as a symptomatic treatment for people with dementia, the evidence regarding particular subgroups, such as younger people, has yet to be fully explored and synthesised in the literature. The aim of this review is to systematically explore and critically appraise the current state of the evidence regarding physical activity for people with young-onset dementia and carers. To achieve the overall aim, this review will:

1. Systematically search both published and unpublished literature to identify evidence regarding physical activity for people with young-onset dementia and carers.
2. Map key concepts (e.g. intervention type, outcome measures, publication date, geographical location) to gain insight into the extent, range and nature of research activity in this area.
3. Provide a critical narrative of the current evidence, identifying physical activity interventions that work (or not), for whom and in what context, and exploring the role of carers’ in such interventions.
4. Identify questions for future research regarding physical activity for people with young-onset dementia and carers.

## Methods

A scoping review will be undertaken to map the extent, range and nature of research in this area. Scoping reviews are particularly useful when exploring emergent research areas, where it is anticipated that evidence will be sparse and heterogeneous, and thus not amenable to more traditional types of systematic review (e.g. meta-analyses) [20]. Scoping reviews seek to provide an overview of all available evidence in a particular field and do not exclude studies based on study design or quality [21]. This was considered useful in the current study as evidence will be sought from unpublished sources, which may not meet rigorous academic standards but could nonetheless provide useful information and insights. Moreover, unlike more traditional reviews which tend to focus on efficacy, this review will seek to explore why certain interventions might work (or not), with specific people, in certain contexts [22]. Elucidating such information would be useful for practitioners devising physical activity interventions in applied settings. The findings of this review could serve as a basis for consultation with key stakeholders, such as younger people with dementia, carers and service providers, and thus could generate collaborative ideas about what would make for effective future physical activity interventions.

The final review output will adhere to the Preferred Reporting Items for Systematic Reviews and Meta-Analysis (PRISMA) statement [23]. Accordingly, this review protocol was developed using the PRISMA-Protocols (PRISMA-P) 2015 checklist [24] (Additional file 1: Table S1). In line with the scoping review methodology, this review will be iterative in nature, and should any amendments be made to the protocol, these will be reported explicitly (i.e. date of amendment, description of the change and rationale for change) in the final review output. At present, scoping reviews are not eligible for registration on the international prospective register of systematic reviews (i.e. PROSPERO). Institutional ethics approval for this review was granted by the Health and Wellbeing Faculty Research Ethics Committee at Sheffield Hallam University (Ref No HWB-2017-18-S&E-03).

### Eligibility criteria

The aim of this review is to systematically explore and critically appraise the current state of the evidence regarding physical activity for people with young-onset dementia and carers. To meet this aim, any evidence that meets the following PICOCS (population, intervention, comparison, outcomes, context, study design) criteria [25] will be included in the review:

#### Population

Adults with young-onset dementia and/or carers. For the purpose of this review, a person with young-onset dementia will be defined as an individual with onset of dementia symptoms before the age of 65 years [26]. Papers reporting participants aged 65 years and older who were diagnosed with dementia before the age of 65 years will be included. No restriction will be placed on type or severity of dementia. A carer will be defined as any individual that supports a person living with dementia in either a formal (i.e. paid) or informal (i.e. unpaid) capacity.

#### Intervention

Any physical activity intervention or programme (e.g. walking, swimming, dance). Physical activity will be defined as ‘any bodily movement produced by skeletal muscles that results in energy expenditure’ [27]. The terms ‘exercise’ and ‘physical activity’ are often used interchangeably within the literature; therefore, this review will seek to be inclusive of all activities provided that they are deemed to be intentional and purposeful (i.e. conditioning exercises, sports/games) but will not include physical activity as a result of daily living (i.e. housework, occupation). Complex interventions or programmes with a physical activity component will be included. Interventions or programmes that report mixed population data (i.e. both young- and late-onset dementia) will be included if data can be separated.

#### Comparison

No comparator required.

#### Outcome(s)

Papers that report any outcome related to the health of the person living with dementia, or carer (e.g. behavioural, cognitive, functional, biomarker, social, activities of daily living, quality of life) and/or papers that report any outcome related to the intervention or programme (e.g. intervention type, outcome measures used, proposed mechanisms of action, underlying theories, adherence, perceived strengths/limitations).

#### Context

No restriction will be placed on participant living circumstances, country of origin or publication date. For pragmatic reasons, searches will be limited to papers published in the English language.

#### Study design

Any study design (i.e. quantitative, qualitative or mixed methods). Programme evaluations and reports will be included. Non-peer reviewed journal articles, opinion pieces, books, book reviews, commentaries, letters and editorials will be excluded. Review articles will be excluded, but relevant papers will be used to crosscheck for primary papers. Personal blogs and social media will be excluded.

### Information sources

Key search terms and, where available, controlled vocabulary terms will be inputted into the electronic databases as follows: MEDLINE (EBSCO), SPORTDiscus (EBSCO), CINAHL (EBSCO), Cochrane Library (Wiley), PsycINFO (ProQuest), Applied Social Sciences Index & Abstracts (ASSIA) (ProQuest) and Scopus (Elsevier). Grey literature will be sought by searching NICE Evidence Search (UK) and targeted international organisations, e.g. Alzheimer’s Society (UK), Age UK, Young Dementia UK, Alzheimer’s Association (USA), Dementia Australia, to source information from a national and international perspective. Three trial registers will be searched: UK Clinical Trials Gateway, International Clinical Trials Registry Platform and EU Clinical Trials Register. Reference lists from papers included in the review will be screened to identify further relevant studies. A bibliography of included literature will be circulated to the review team for consideration. Approximately five to ten (based on availability and responsiveness) internationally recognised experts in the field of dementia and physical activity will be contacted via a maximum of two emails to offer insight and input. The principal investigator (CR) will contact specialist interest groups such as Sheffield Hallam University; Dementia research cluster, University of Bradford; Centre for Applied Dementia Studies, Dementia Action Alliance (UK) and Alzheimer’s Disease International to enquire about pertinent sources of literature available for the review. Responses will be shortlisted and discussed amongst the review team to ensure national and international coverage. This will be further supplemented by internet searches to identify any key stakeholders/organisations that may have been missed during the initial consultation exercise. Dates of the initial and final search will be reported in the final output.

### Search strategy

Key search terms will be discussed and agreed by the principal investigator (CR) and an information scientist (DH) with over 12 years of experience of undertaking systematic searches. The search will likely include two facets: (1) terms to describe young-onset dementia and (2) terms to describe physical activity, with appropriate synonyms for each facet. The search strategy will be developed by CR and DH, with intellectual input from the review team (DP, DR and JS). A draft MEDLINE search strategy will be devised and piloted by CR and DH. The final MEDLINE search strategy will be adapted to suit the syntax and subject headings of other databases used in this review. Searches will not be limited by date.

### Data management

All search results will be exported to Refworks (ProQuest, 2017), an online reference management system. Refworks will be used to delete duplicate records. Microsoft Excel (Microsoft Corporation, 2010) will be used to support the screening and data extraction process.

### Study selection

Study selection will be undertaken in three stages. First, a pilot exercise will be undertaken to assess the inter-rater reliability of applying the eligibility criteria. Second, titles and abstracts will be screened against the eligibility criteria. Third, the full text of any remaining papers will be screened in order to determine whether the article should be included in the review. If necessary, the principal investigator (CR) will seek additional information from the corresponding author to resolve queries about eligibility (via a maximum of two emails). The principal investigator (CR) will undertake all selection stages, and 10% of papers will be double screened by a second reviewer (DR). Disagreements between the two reviewers will be discussed until a consensus is reached. Should reviewers not reach a consensus, a third reviewer (DP) will be consulted. Reviewers will not be blinded to the journal title, study authors or associated institutions. A PRISMA flow diagram will be presented in the final output to show the search and screening processes.

### Data extraction

A data extraction form will be developed by CR and DH a priori and will be published as an appendix in the final review output. Prior to the formal data extraction, a pilot extraction will be undertaken by CR and verified by DH to check for errors in extraction and appropriateness of the extraction form. Data from full-text articles will be extracted by one author (CR), and a proportion will be verified by another (DR) to reduce risk of error in data extraction. Any disagreements between the reviewers will be discussed until a consensus is reached. Should reviewers not reach a consensus, a third reviewer (DP) will be consulted. In the event that multiple papers use one dataset, this will be made explicit in the final report. If data is missing, unclear or presented in a way that is unsuitable for this review, CR will contact the corresponding study author for appropriate data (via a maximum of two emails). Any non-responses will be documented and reported anonymously in the final report.

Data will be extracted using a standardised data extraction form and will likely include:

1. Study characteristics—title, author(s), publication year, place of publication, study aims/objective(s)/research question(s), study type, sample size, inclusion/exclusion criteria, recruitment method, data collection method, data analysis method, country where study was undertaken, study date and duration (incl. follow-up) and funding source/body.
2. Participant characteristics—demographics (age, sex, ethnicity), dementia type, dementia severity, time since diagnosis, current living circumstance, carer demographics (age, sex, ethnicity), carer relationship to the person with dementia and time commitment to caring (e.g. part-time, full-time).
3. Intervention characteristics—intervention/programme type (e.g. walking, swimming, dance), description (e.g. structured vs. unstructured, individual vs. group), session duration (i.e. total number of minutes), intensity of activity (e.g. low/moderate/high), frequency (i.e. number of sessions per week), total duration of intervention/programme (i.e. number of weeks delivered), setting (e.g. leisure centre, day care centre), intervention/programme specificity (i.e. dementia specific or open to the wider community), deliverer characteristics (e.g. occupational therapist, activity coordinator), involvement of carer in intervention/programme (i.e. yes/no), given rationale for the intervention/programme (e.g. enjoyment, social interaction, mobility), feasibility/evaluation measures (e.g. recruitment, adherence), type of comparator used (if any) (e.g. standard/usual care, sham exercise), geographical location of intervention/programme, impact (i.e. international, national or local), sector (i.e. public, private, referral only) and cost to service user.
4. Outcome characteristics—outcome type (e.g. behavioural, cognitive, functional, biomarker, feasibility), method or instrument used to collect outcome data, patient or proxy-reported outcomes, frequency of outcome measurement, author conclusions, recommendations for future research and study limitations.

During the data extraction stage, the principal investigator (CR) will arrange and chart key pieces of information extracted from each paper to map the extent, range and nature of the research in this area. The charting exercise will be iterative in nature, enabling the principal investigator to group raw data into initial themes and areas for further exploration in the data synthesis stage. The review team (DP, DR, JS) will be consulted at various stages throughout the charting exercise to ensure that the data items extracted are consistent with the overall aim of the review [28].

### Quality assessment

In line with the aim of a scoping review, to include all available evidence, studies will not be excluded based on quality [21]. However, the methodological quality of individual studies included in the review will be assessed using an appropriate Joanna Briggs Institute (JBI) critical appraisal tool [29] to inform discussion around the overall ‘strength of the evidence’ in this area. Grey literature sources will also be appraised using the Authority, Accuracy, Coverage, Objectivity, Date, Significance (AACODS) checklist [30].

### Data synthesis

It is anticipated that included studies will be heterogeneous, and thus, the possibility of meta-analysis will be low. Quantitative, qualitative and mixed methods findings will therefore be subjected to a narrative synthesis. The information will be presented in a tabular and text format to highlight the study characteristics and key research findings of the included studies. In line with the general aim of a scoping review, to map out the research landscape, some form of visual representation of the data will be presented in the results section to map the extent, range and nature of research in this area. The narrative synthesis, guided by Popay et al. [31], will seek to explore similarities and differences, both within and between studies, to identify patterns and themes and postulate explanations for findings (i.e. how and why certain interventions or programmes have worked (or not); factors that might have influenced the findings). Subgroup analysis may be undertaken, if the data allows and could include comparison of different intervention/programme characteristics (e.g. group vs. individual physical activity, structured vs. unstructured), different participant characteristics (e.g. mild vs. moderate vs. severe dementia or male vs. female) and different dementia types (e.g. Alzheimer’s disease vs. frontotemporal dementia). The narrative synthesis will also consider the robustness of the synthesis itself by reporting on the overall strength and confidence of the findings and, where possible, at individual finding level. Data synthesis will be undertaken by the principal investigator (CR) and discussed amongst the review team (DP, DR, JS) for validation.

## Discussion

To the knowledge of the authors, this will be the first review to systematically explore and critically appraise the current state of research evidence regarding physical activity for people with young-onset dementia and carers. It is anticipated that the findings from this review will help to inform the development of future physical activity interventions or programmes for people with young-onset dementia and carers and that the information gathered might be of interest to academics, practitioners, people living with young-onset dementia and carers.

## Additional file

Additional file 1: **Table S1.** PRISMA-P (Preferred Reporting Items for Systematic review and Meta-Analysis Protocols) 2015 checklist: recommended items to address in a systematic review protocol*. (DOCX 23 kb)

## Abbreviations

AACODS: Authority, Accuracy, Coverage, Objectivity, Date, Significance
ASSIA: Applied Social Sciences Index & Abstracts
CINAHL: Cumulative Index to Nursing and Allied Health Literature
MEDLINE: Medical Literature Analysis and Retrieval System Online
PICOCS: Population, intervention, comparison, outcomes, context, study design
PRISMA: Preferred Reporting Items for Systematic Reviews and Meta-Analysis
PRISMA-P: Preferred Reporting Items for Systematic Reviews and Meta-Analysis Protocols
PROSPERO: International Prospective Register of Systematic Reviews
